# Granulomatous lobular mastitis involving accessory breast tissue: a case report and review of the literature

**DOI:** 10.3389/fonc.2026.1960013

**Published:** 2026-10-20

**Authors:** Xiao-Fa Li, Shan-Shan Meng, Tang-Shun Wang, Xi-Meng Zuo, Xiang Gao, Xiao-Guang Shi

**Affiliations:** Department of General Surgery, Dongzhimen Hospital of Beijing University of Chinese Medicine, Beijing, China

**Keywords:** accessory breast tissue, breast tissue excision, diagnosis, granulomatous lobular mastitis, treatment

## Abstract

Granulomatous lobular mastitis (GLM) is a chronic inflammatory disease of the breast. Although its incidence has increased in recent years, GLM involving accessory breast tissue has rarely been reported. This study describes the complete clinical course of a patient with GLM involving accessory breast tissue, including disease onset, diagnosis, and treatment. The patient presented with persistent swelling, pain, and ulceration of a left axillary mass. Multiple courses of antibiotic therapy failed to produce significant clinical improvement. Based on the clinical manifestations, GLM involving accessory breast tissue was considered the most likely diagnosis. Histopathological examination following surgical treatment ultimately confirmed the diagnosis of GLM. This review aims to consolidate the available evidence regarding the clinical presentation, diagnosis, and management of GLM involving accessory breast tissue and provide a reference for its diagnosis and management.

## 1 Introduction

Granulomatous lobular mastitis (GLM) is a chronic inflammatory breast disease involving breast lobules, characterized by lobulocentric, non-caseating and frequently suppurative granulomatous inflammation. First reported by Kessler et al. in 1972, the incidence of GLM has increased markedly in recent years (1). The condition predominantly affects women of reproductive age with a history of pregnancy and lactation. Although its etiology remains incompletely understood, proposed contributing factors include autoimmune responses, hyperprolactinemia, microbial infection, and oral contraceptive use. Clinically, GLM typically presents as a breast mass accompanied by erythema and localized swelling and may subsequently progress to abscess formation, sinus tract development, and ulceration with purulent discharge. Owing to its prolonged clinical course and overlapping clinical features with inflammatory breast carcinoma, the risk of misdiagnosis remains high. Current treatment strategies include medical therapy, such as glucocorticoids, immunosuppressive agents, and antibiotics, as well as surgical excision; however, no consensus has been established regarding the optimal management approach.

Accessory breast tissue is a congenital developmental anomaly resulting from incomplete regression of the embryonic mammary ridge. It has an estimated prevalence of 2%–6% and most commonly occurs in the axillary region. GLM involving the accessory breast tissue is a benign chronic inflammatory condition involving the lobular units of ectopic mammary tissue. The present study describes a patient with GLM involving accessory breast tissue treated at Dongzhimen Hospital, Beijing University of Chinese Medicine. The clinical characteristics, diagnostic evaluation, and treatment course of these cases were reviewed and integrated with the available published literature to summarize the current evidence regarding the diagnosis and management of this condition, thereby providing information that may assist clinical decision-making.

## 2 Case presentation

Patient information: A 32-year-old female presented with a 2-month history of painful left axillary mass.

History of present illness: The mass arose without obvious precipitating factors, accompanied by local pain and overlying skin erythema, and gradually grew to egg-size. The patient received one-week intravenous levofloxacin and ofloxacin therapy at a local hospital without obvious symptom improvement. She subsequently underwent intermittent intravenous anti-inflammatory treatment (drug details unknown), followed by repeated local injections of gentamicin sulfate plus triamcinolone acetonide acetate, which achieved partial pain relief but with intermittent symptom relapse. Intermittent cephalosporin antibiotics were also administered, yet her left-axilla-related pain progressively worsened. She was then referred to our breast surgery outpatient clinic.

Past medical and surgical history: She had undergone bilateral axillary fibroma excision and right accessory nipple excision 13 years previously; two cesarean-section deliveries occurred 5 years and 1 year prior to admission, and cholecystectomy was performed 1 year ago.

Personal, obstetric and lactation history: Occasional alcohol intake was reported; oral contraceptive use was denied. She had two full-term pregnancies with lactation history, and no remarkable allergy history.

Review of systems: No fever, headache, dizziness, chest distress, arthralgia or erythema of lower extremities. Appetite and bowel-bladder function were normal; only sleep quality was poor. No significant body-weight change.

Physical examination: Vital signs were stable. Cardiopulmonary and abdominal examinations were unremarkable. Bilateral breasts were symmetrical, without nipple-areola deformity or peau-d’ orange change; no palpable breast mass.

Right axilla showed two well-healed surgical scars (10 cm transverse, 4 cm longitudinal). No supra-/infra-clavicular lymphadenopathy on the right side.

Left axilla demonstrated a 10-cm transverse old surgical scar. Beneath the scar laid an indurated mass sized approximately 8.0 × 6.0 cm with ill-defined margin, associated with local erythema, swelling (5.0 × 3.0 cm), tenderness and elevated skin temperature (**Figure 1**). Two small 0.3-cm ulcerative lesions above the scar discharged yellow purulent exudate. Several palpable enlarged left axillary lymph nodes (max 1.0 × 1.0 cm); no left supra- or infra-clavicular lymphadenopathy.

**Figure 1:**
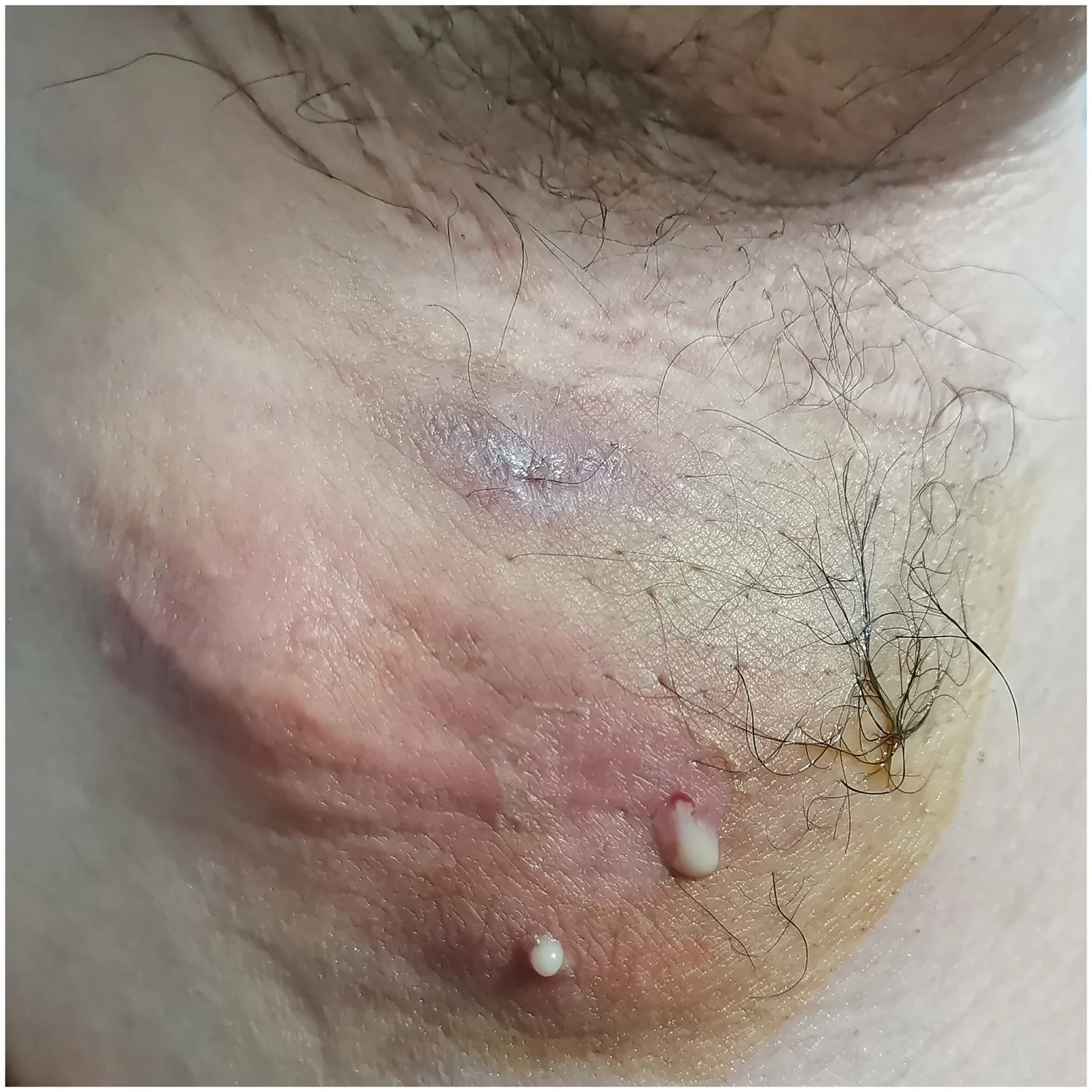
Preoperative appearance of the left accessory breast.

Imaging findings: Repeat breast ultrasonography (**Supplementary Figures 1A,B**): bilateral axillary accessory-gland tissue was detected. On the right side, a 1.1 × 0.5 cm mixed-echogenic nodule (BI-RADS 3) was seen within accessory breast tissue. On the left side, multiple patchy mixed-echogenic lesions (maximum 4.1 × 3.4 × 1.0 cm) with ill-defined margins, scattered punctate hyperechoic foci and abundant surrounding blood flow were noted, consistent with inflammatory lesion combined with abscess formation. Mild enlargement of left axillary lymph nodes was also observed.

Based on clinical manifestations, physical examination and ultrasonography, GLM involving left-sided accessory breast tissue was highly suspected, and surgical intervention was recommended. After completing preoperative assessment without surgical contraindications, the patient received bilateral accessory breast tissue excision combined with local skin-flap transfer and reconstruction under general anesthesia.

Surgical procedure: On the right side, a 3-cm fusiform skin incision was made along natural skin-tension lines. After flap elevation, the right accessory breast tissue was completely excised along the superficial layer of cribriform fascia. A drainage tube was placed after irrigation, and subcutaneous tissue and skin were closed with interrupted sutures.

On the left side, a 5-cm fusiform incision plus an additional 2-cm triangular extension was performed over swollen and ulcerated skin. Multiple abscess cavities filled with inflammatory necrotic granulation tissue were exposed after subcutaneous dissection. Yellowish caseous lipid-like substance extruded from ducts under gentle compression, indicating extensive inflammatory involvement of left accessory breast tissue. The left accessory breast tissue was fully excised. Gross examination of the surgical specimens is shown in **Supplementary Figures 2A,B**. A lateral thoracic adipofascial flap was harvested to repair the axillary defect for contour restoration and satisfactory cosmetic outcome. The operative field was thoroughly irrigated; closed-suction drainage was placed via a separate stab incision, followed by layered interrupted suture closure. Wounds were dressed with gauze bolster and moderate-pressure elastic bandage.

Post-operative course: Post-operative supportive treatment including anti-infective therapy, analgesia and fluid infusion was administered. Drainage volume was monitored daily. Drainage output was 10 mL (POD 1), 10 mL (POD 2), zero output on POD 3; the drain was removed on POD 4 (**Figure 2A**). Bilateral axillary incisions healed well; sutures were removed on POD 14 before discharge (**Figure 2B**).

**Figure 2:**
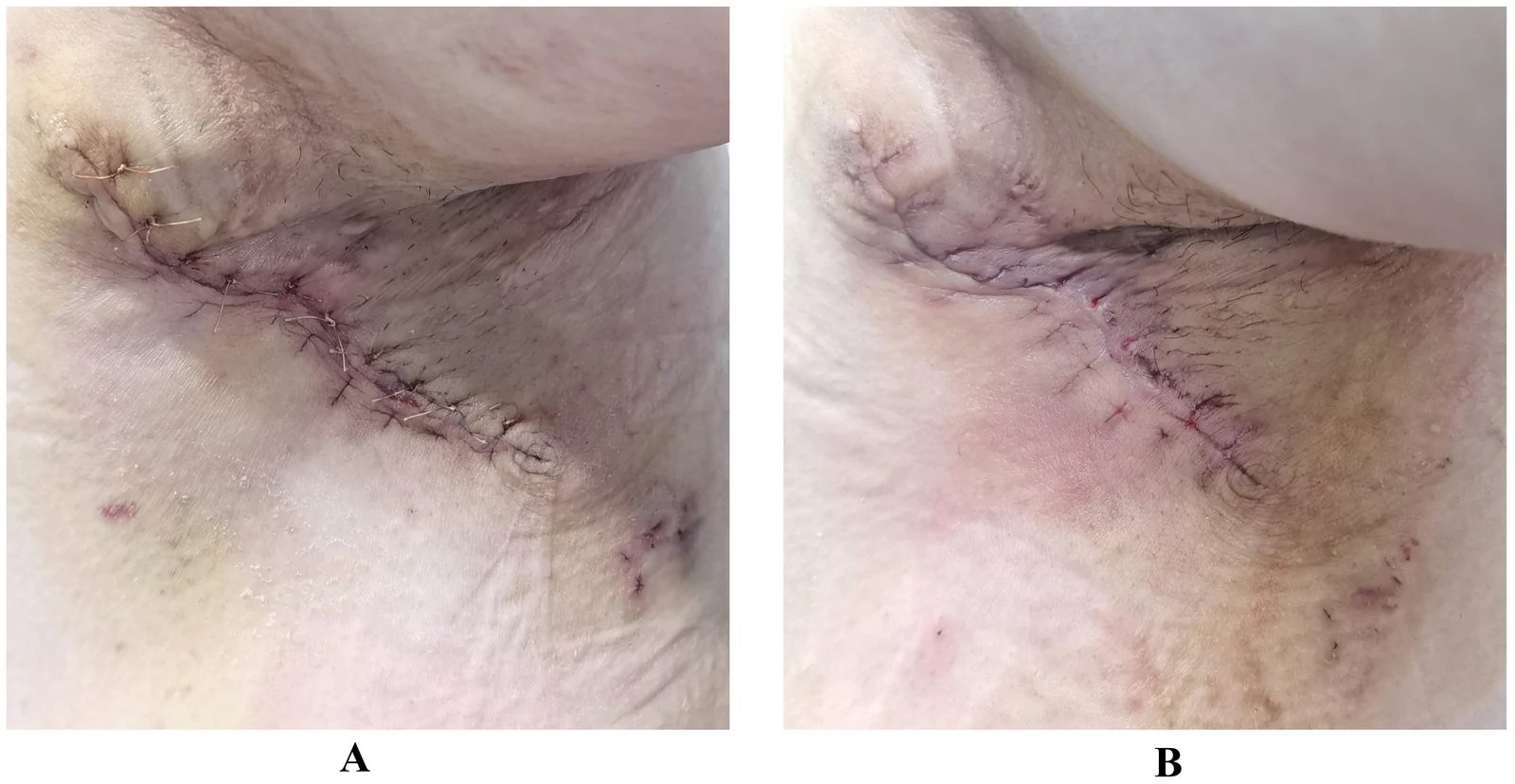
Postoperative appearance of the left accessory breast at different time points. **(A)** On POD4, an approximately 8-cm surgical incision was visible in the left axilla, with the drainage puncture wound located inferior and posterior to the incision. **(B)** On POD14, the surgical incision had healed well. After suture removal, the skin at the drainage puncture wound located inferior and posterior to the incision had almost completely healed.

Histopathological results: The left-side specimen confirmed granulomatous lobular mastitis with duct ectasia (**Figures 3A, B**); the right-side specimen showed isolated duct ectasia without GLM (**Figures 3C, D**).

**Figure 3:**
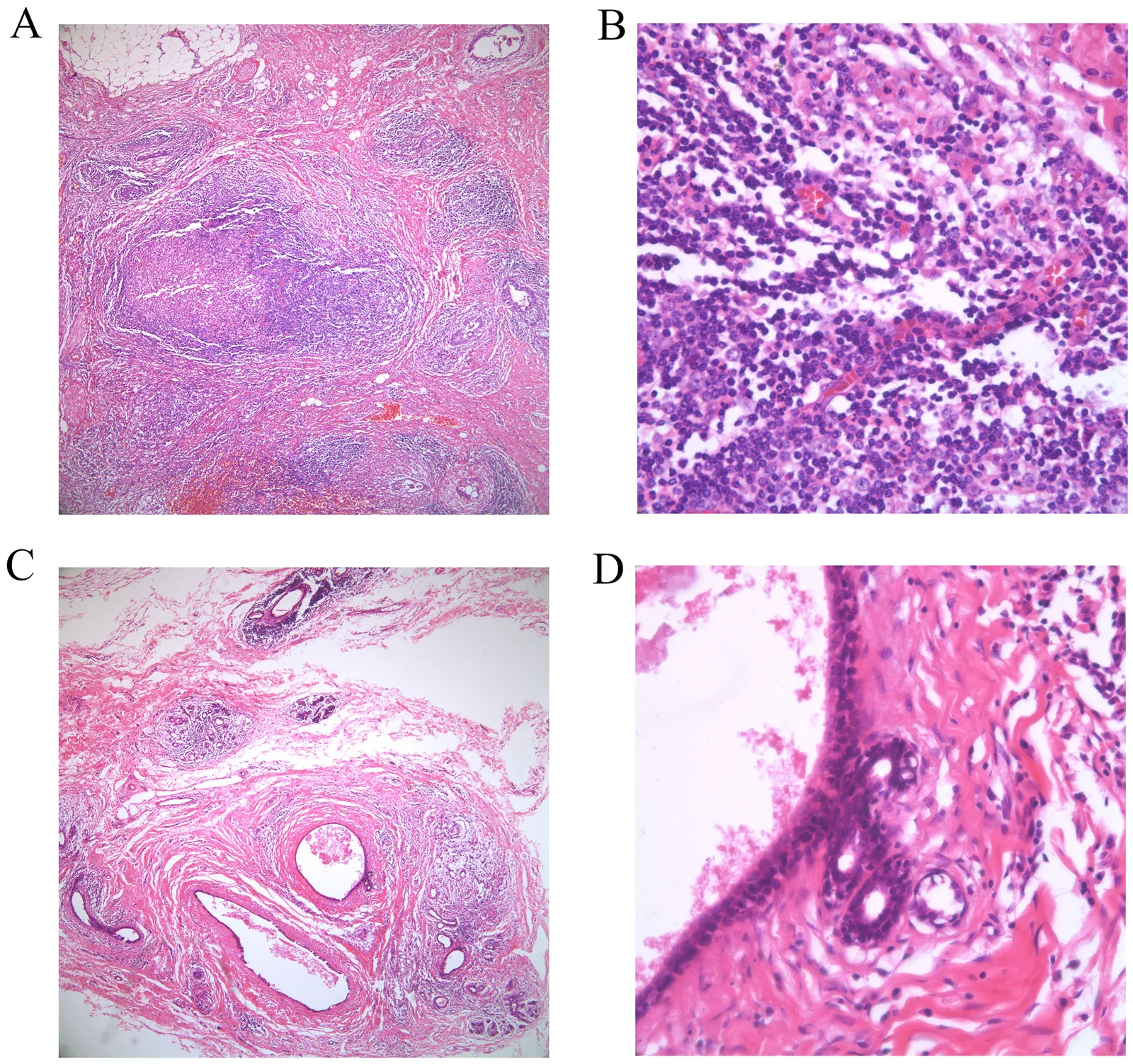
Histopathological images of the bilateral accessory breast tissue. **(A)** Low-power microscope (×40): Left lateral accessory breast tissue image shows a granulomatous inflammatory distribution pattern centered around the lobules, with the lesion surrounding the terminal ductular units. **(B)** High-power magnification (×400): Left lateral breast tissue image shows granulomatous structure, accompanied by extensive infiltration of neutrophils, lymphocytes, plasma cells, and epithelioid cells, as well as lipid vacuoles and fat necrosis. **(C)** Low-power microscope (×40): Right lateral breast tissue show dilation of breast ducts. **(D)** High-power magnification (×400): Right lateral breast tissue show dilation of the breast ducts.

Follow-up: At 1-year post-operative follow-up, repeat breast ultrasonography revealed no abnormalities (**Figure 4**).

**Figure 4:**
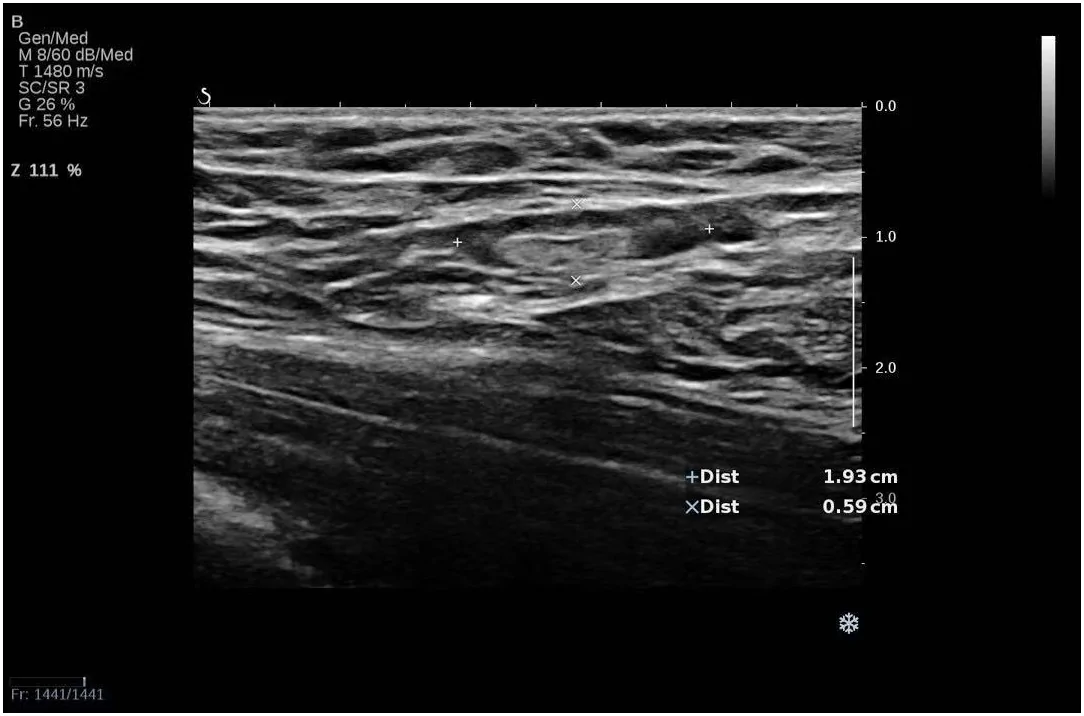
The ultrasound results at the one-year follow-up visit.

## 3 Literature search

For this case report, a narrative literature review was performed. We searched PubMed, Web of Science, Embase, CNKI and Wanfang Data from database inception to March 1st, 2026. Search terms included “granulomatous lobular mastitis” OR “granulomatous mastitis” combined with “accessory breast”, “axillary breast”, “supernumerary breast”, “ectopic breast”, with corresponding Chinese terms for Chinese-language databases. Publications were limited to English and Chinese. Eligible reports required histopathologically-confirmed granulomatous lobular mastitis arising within accessory/ectopic breast tissue. This is a narrative rather than a systematic review, so exhaustive literature retrieval cannot be guaranteed. A review of the Chinese and international literature identified only five reported cases of GLM involving accessory breast tissue, highlighting the extreme rarity of this condition.

## 4 Discussion

### 4.1 Overview of accessory breast tissue

Accessory breast tissue is a congenital anomaly that results from incomplete regression of the embryonic mammary ridge during fetal development. During embryonic development, the mammary primordia arise from localized ectodermal epithelial thickening along the mammary ridge. Failure of regression of these structures leads to the formation of accessory breast tissue (2). Approximately 2%–6% of women have accessory mammary glands or axillary breast tissue, and a familial predisposition has been reported in approximately 1%–5% of cases (3).

Accessory breast tissue may develop at multiple sites along the embryonic milk line, most commonly in the axillary region, although it can also be found on the thoracic wall and abdomen. Less frequently, it is identified on the face, neck, or upper extremities. As physical maturation progresses and adipose tissue accumulates, accessory breast tissue may gradually enlarge. Consequently, related symptoms often become clinically apparent after weight gain during adulthood.

Accessory breast tissue is commonly classified according to the Kajava classification system based on the presence or absence of glandular tissue, the nipple, and the areola: (1) glandular tissue, nipple, and areola present; (2) glandular tissue and nipple present; (3) glandular tissue and areola present; (4) glandular tissue only; (5) nipple and areola only (pseudomamma); (6) nipple only; (7) areola only; (8) hair only. Clinically, the most common presentation is incomplete accessory breast tissue containing glandular tissue alone (4).

In the present case, the patient had previously undergone excision of the right accessory nipple, while glandular tissue remained in both axillae. Prior to excision of the accessory nipple, the right accessory breast tissue would have corresponded to Kajava class II. Because accessory breast tissue shares the same histological characteristics as orthotopic breast tissue, it is similarly influenced by endocrine hormonal changes. Consequently, physiological and pathological changes may occur during the menstrual cycle, pregnancy, and lactation. Ryul et al. (5) reported that pregnancy may increase the volume of both adipose and glandular tissue within accessory breasts and that lactation can occur in accessory breast tissue after childbirth. Although accessory nipples and glandular tissue were present before surgery in the current patient, milk secretion from the accessory breast tissue had not been observed before disease onset.

To date, no studies have demonstrated an association between milk secretion in accessory breast tissue and the presence of accessory nipples. Further investigation is therefore warranted to clarify the relationship between the anatomical characteristics of accessory breast and the development of disease. Accessory breast tissue is susceptible to many of the same pathological conditions as orthotopic breast tissue. In addition to inflammatory disorders, both benign and malignant neoplasms may arise in accessory breast tissue. Among these conditions, carcinoma is the most frequently reported, followed by inflammatory lesions, fibroadenoma, phyllodes tumor, and fibrocystic changes (6). GLM involving accessory breast tissue has been reported only rarely. Therefore, the following section reviews the published literature on GLM involving accessory breast tissue in the context of the present case.

### 4.2 Summary of clinical features

GLM is a benign chronic inflammatory breast disease first described by Kessler et al. in 1972 (1). The prevailing hypothesis attributes its pathogenesis to hypersensitivity-mediated autoimmune inflammation. Common risk factors include history of pregnancy and breastfeeding, recent pregnancy, hyperprolactinemia, oral contraceptive use, α1-antitrypsin deficiency, breast trauma, and microbial infections—particularly those caused by Corynebacterium species (7, 8). Histopathologically, GLM presents as non-caseating granulomatous inflammation centered around terminal duct lobular units, often accompanied by suppurative granulomas with lipid vacuoles at their centers and infiltration of various inflammatory cells (9).

Due to the scarcity of reported cases involving accessory mammary tissue and the lack of standardized diagnostic criteria, we analyzed existing case reports to provide insights that may serve as clinical reference for diagnosis.

1. Clinical features: Disease onset may occur without obvious triggers, although some patients have a history of trauma to accessory breast tissue. The condition primarily affects parous women with a history of breastfeeding, most commonly within 3–5 years after childbirth. This patient had prior breastfeeding experience; physiological fluctuations in prolactin, estrogen, and progesterone levels during lactation may stimulate residual accessory breast tissue, leading to glandular hyperplasia and ductal dilation.
2. Anatomical features: The disease appears more frequently in patients with accessory nipples. In this case and most published cases, the accessory breast tissue belongs to Kajava class II—characterized by the presence of both glandular tissue and nipple structures. Accessory nipples are often associated with short subcutaneous duct systems whose openings are small or underdeveloped, allowing secretions to accumulate distally in ducts or lobules, potentially promoting ductal dilation and secretion retention, thereby initiating sterile granulomatous inflammation.
3. Clinical presentation and physical examination: The disease typically manifests as a painful mass within the accessory breast tissue, sometimes accompanied by erythema, swelling, abscess formation, or ulceration with purulent discharge. Lesions are usually firm or board-like, with irregular contours and poorly defined borders, often tender to palpation. The mass may rapidly become purulent, and fluctuance may be palpable when an abscess forms. Regional lymphadenopathy on the same side may also occur. Some cases present systemic manifestations such as erythema nodosum or arthritis, but this patient showed no systemic symptoms, and preoperative immunological evaluation was unremarkable.
4. Imaging findings: Imaging features are relatively nonspecific. Ultrasound remains the primary modality for diagnosis and differential diagnosis, helping assess lesion extent to guide incision and drainage or surgical intervention. GLM typically appears as an ill-defined, irregular hypoechoic lesion, possibly with ductal dilation. Color Doppler imaging often reveals increased vascularity in areas of active inflammation or abscess formation.
5. Histopathological findings: Histopathological examination remains the gold standard for diagnosing GLM. Characteristic findings include non-caseating granulomatous inflammation centered around terminal duct lobular units, with abundant epithelioid histiocytes and multinucleated giant cells forming well-circumscribed granulomas detected by fine-needle aspiration cytology. Lipid vacuoles are commonly seen at the center of granulomas, surrounded by dense inflammatory infiltrates dominated by neutrophils, along with varying degrees of fat necrosis (10).

Due to its nonspecific clinical presentation, GLM is often mistaken for inflammatory breast cancer (IBC) or periductal mastitis (PDM). Key differentiating features are as follows (11):

1. Differentiation from IBC: Both GLM and IBC can present with breast erythema, edema, fever, pain, and palpable masses, which is why GLM is sometimes referred to as a “pseudocarcinomatous” condition. On ultrasound, IBC typically shows skin thickening, diffuse increased echogenicity of breast parenchyma, ill-defined lesions, and prominent intratumoral vascularity. However, definitive diagnosis relies on histopathology via core needle or fine-needle biopsy, where identification of malignant cells defines IBC (12).
2. Differentiation from PDM: PDM typically involves the nipple-areolar complex, whereas GLM mainly affects peripheral terminal duct lobular units. In accessory breast tissue, distinguishing between the two based solely on lesion location is often difficult, as accessory breasts are typically small and have underdeveloped nipples and areolae; therefore, histopathological examination of tissue obtained by needle biopsy or surgical excision is crucial. Unlike GLM, which is characterized by lobular-centered granulomatous inflammation, PDM usually presents with mild dilation of minor ducts accompanied by dense periductal infiltration of lymphocytes and plasma cells (13).
3. Deferential Diagnosis: Furthermore, the differential diagnosis should also include other inflammatory and granulomatous breast diseases such as IgG4-related mastitis, tuberculous mastitis, fat necrosis, and foreign body granuloma (14).

### 4.3 Treatments

A variety of treatment strategies are currently available for GLM; however, no consensus has been established regarding the optimal management approach. Current treatment options can generally be categorized as conservative medical therapy or surgical intervention. Medical management includes corticosteroids, antibiotics, immunosuppressive agents, and traditional Chinese medicine.

Given the proposed etiological role of *Corynebacterium* species in the pathogenesis of GLM, several studies have suggested that lipophilic antibiotics, such as trimethoprim and clindamycin, may be effective in the treatment of GLM (15). Topical corticosteroids are also considered a first-line therapeutic option for many patients (16). In addition, immunosuppressive agents, such as methotrexate, azathioprine, and cyclosporine A, have demonstrated therapeutic efficacy in selected patients with GLM (17, 18). However, medical therapy may be associated with adverse reactions, including Cushing syndrome, weight gain, and hyperglycemia.

Surgical management primarily includes incision and drainage for abscesses and complete surgical excision of the affected tissue. Rotational gland dissection has been reported as an effective surgical approach for refractory granulomatous mastitis. In a single-center retrospective study of 129 patients with refractory granulomatous mastitis, rotational gland dissection achieved a recurrence rate of 3.10% at a median follow-up of 56 months (19). Importantly, this technique combines extensive exposure and removal of the involved tissue with preservation/reconstruction of the surrounding breast tissue, supporting the principle that adequate disease clearance can be achieved while maintaining satisfactory cosmetic outcomes. Although that cohort was not specifically limited to accessory-breast GLM, it provides useful evidence regarding the principles and outcomes of surgical treatment for refractory granulomatous mastitis, and supports our individualized approach of complete excision of the involved tissue with flap reconstruction for GLM involving accessory breast tissue. In clinical practice, a multidisciplinary treatment strategy is frequently adopted, in which traditional Chinese medicine, antibiotics, or corticosteroids are administered preoperatively to control inflammation and reduce the extent of localized lesions before surgery is performed based on the patient’s clinical condition (20).

Because only a limited number of cases of GLM involving accessory breast tissue have been reported, evidence regarding the optimal treatment strategy remains insufficient. Anıl et al. (6) and Jun et al. (21) each reported a case of granulomatous mastitis involving accessory breast tissue during pregnancy. In the case reported by Anıl et al. (6), the patient was managed conservatively with local intralesional triamcinolone acetonide injections. In contrast, Jun et al. (21) described a patient who underwent incision and drainage of a local abscess after antibiotic therapy failed to achieve clinical improvement, followed by surgical excision one week after delivery. Guo et al. (16), Ding et al. (22), and Liu et al. (23) performed surgical excision after local inflammation had been adequately controlled with medical therapy.

Based on the currently available literature and the present clinical experience, a treatment strategy combining preoperative medical therapy with subsequent surgical intervention appears to be a reasonable approach for selected patients with GLM involving accessory breast tissue. Further evidence is needed before standardized diagnostic and treatment recommendations can be established.

Before referral to our institution, the patient had received multiple courses of systemic antibiotics and corticosteroid therapy for the left axillary mass. These treatments provided limited clinical benefit while imposing a considerable physical burden on the patient. Approximately 40 days after the onset of the disease, the lesion progressed to ulceration with purulent discharge. Despite the extensive inflammatory involvement, definitive surgical excision rather than local debridement and drainage was selected after consideration of the lesion characteristics, the treating team’s experience, and the patient’s preferences. The objectives were to shorten the disease course, alleviate pain, and reduce the psychological burden and cutaneous damage associated with recurrent disease.

Preoperatively, ultrasonography revealed a complex cystic and solid nodule (BI-RADS category 3) in the right axillary accessory breast tissue. The right-side procedure was performed at the patient’s request, based on individualized surgical decision-making, to address the BI-RADS 3 lesion and to reduce the potential need for future treatment and surveillance of the contralateral side. Final histopathology of the right specimen demonstrated duct ectasia without GLM. We emphasize that this decision reflected patient preference and individualized surgical judgment rather than an evidence-based indication, and that routine prophylactic excision of unaffected contralateral accessory breast tissue is not supported by currently available evidence; such decisions should be individualized and made after full discussion of the risks and benefits with the patient.

Simple excision of the palpable mass alone was considered insufficient because of its irregular shape and ill-defined borders. Complete excision of the involved inflammatory tissue, abscesses, dilated ducts and affected skin, together with an appropriate rim of adjacent normal breast tissue when necessary, was performed to reduce the risk of postoperative recurrence. Because GLM is an inflammatory rather than a malignant disease, the objective and extent of surgery should be tailored to the individual lesion rather than aimed at achieving histologically negative resection margins as an absolute requirement. Local flap reconstruction was subsequently performed to restore the contour and appearance of the surgical site. This approach may be particularly important for accessory breast lesions, because these lesions usually occur in a relatively confined and cosmetically sensitive region, where complete excision of the lesion combined with appropriate reconstruction can facilitate disease control and satisfactory functional and cosmetic outcomes (24).

## 5 Conclusion

The present report provides a detailed description of a patient with GLM involving accessory breast tissue. In addition, clinical characteristics, diagnostic considerations, and treatment approaches are summarized by integrating the findings from this index case with the available published literature.

As GLM involving accessory breast tissue is rare and high-quality evidence remains limited, treatment strategies should be individualized according to disease severity, lesion extent, patient characteristics, and patient preferences. Further clinical studies are warranted to establish evidence-based diagnostic criteria and standardized treatment recommendations for GLM involving accessory breast tissue.

## Data Availability

The original contributions presented in the study are included in the article/**Supplementary Material**. Further inquiries can be directed to the corresponding author.

## Supplementary material

Supplementary Figure 1 Preoperative color Doppler ultrasonography of bilateral accessory breast. **(A)** A mixed-echogenic nodule measuring 1.1 × 0.5 cm was identified in the right accessory breast. The lesion had a regular shape with well-defined margins, with no detectable intralesional or perilesional flow on color Doppler imaging. The elastography score was 2. **(B)** Multiple patchy mixed-echogenic lesions, measuring up to 4.1 × 3.4 × 1.0 cm, were identified in the left accessory breast. The lesions exhibited irregular morphology and poorly defined margins. Large patchy regions of intermediate-to-low echogenicity containing scattered punctate echogenic foci were observed, accompanied by abundant linear vascular flow signals within and surrounding the lesions on color Doppler imaging. The elastography score was 3.

Supplementary Figure 2 Gross appearance of the bilateral accessory breast tissue specimens following surgical excision. **(A)** Gross specimen from the left accessory breast tissue measuring approximately 10.0 × 8.0 cm, showing indistinct glandular tissue with multiple abscess cavities. **(B)** Gross specimen from the right accessory breast tissue measuring approximately 5.0 × 3.0 cm, showing yellowish-white mottled glandular tissue with multiple nodules.
